# A Synbiotic Combination of *Lactobacillus gasseri* 505 and *Cudrania tricuspidata* Leaf Extract Prevents Stress-Induced Testicular Dysfunction in Mice

**DOI:** 10.3389/fendo.2022.835033

**Published:** 2022-04-20

**Authors:** Jae Yeon Joung, Whasun Lim, Yeon Jeong Seo, Jiyeon Ham, Nam Su Oh, Sae Hun Kim

**Affiliations:** ^1^ Department of Biotechnology, College of Life Sciences and Biotechnology, Korea University, Seoul, South Korea; ^2^ Department of Biological Sciences, Sungkyunkwan University, Suwon, South Korea; ^3^ Department of Biotechnology, Institute of Animal Molecular Biotechnology, College of Life Sciences and Biotechnology, Korea University, Seoul, South Korea; ^4^ Department of Food and Biotechnology, Korea University, Sejong, South Korea

**Keywords:** male reproductive function, synbiotics, *Lactobacillus gasseri* 505, *Cudrania tricuspidata* leaf extract, chronic stress

## Abstract

This study investigated the effects of a synbiotic combination (Syn) of *Lactobacillus gasseri* 505 (505) and *Cudrania tricuspidata* leaf extract (CT) on the hypothalamic-pituitary-gonadal axis in mice under chronic stress. Unpredictable chronic mild stress (UCMS) significantly increased the serum levels of corticosterone, however, treatment with Syn suppressed UCMS-induced increases. Histopathological analysis of the testes showed that these organs experienced some damage during UCMS, but this was repaired following treatment with Syn. Similarly, the transcription levels of gonadotropin-releasing hormone (*GnRH*), GnRH receptor, and gonadotropins, moreover, testicular development (i.e., *Adam5*, *Adam29*, and *Spam1*) - and steroidogenesis (i.e., *Lhr*, *Egfr*, and *StAR*) -related genes were significantly downregulated by UCMS. These UCMS-induced changes were inhibited by the administration of Syn, which was confirmed by the results of *in situ* hybridization analysis. These results suggest that the administration of Syn could attenuate the testicular dysfunctions induced by UCMS.

## Introduction

Stress is a widespread condition, with stressors becoming increasingly more prevalent in modern society, with chronic stress causing a number of health problems (1). Chronic stress has a destructive effect on tissues and negatively influencing cellular proliferation and differentiation (2). In addition, many results supported that chronic stress leads to morphological and functional alterations in the testes such as impaired sperm production, reduced serum testosterone levels, and histological alteration (3, 4). Stress-induced increases in serum glucocorticoid concentrations disrupt and suppress endocrine signaling in the male reproductive system *via* the hypothalamic-pituitary-gonadal (HPG) axis resulting in testicular involution (5, 6). Glucocorticoid is described as the stress hormone because its levels rise sharply in response to stress. This sharp increase can result in testicular involution due to a significant drop in pituitary responsiveness to gonadotropin-releasing hormone (GnRH) and the secretion of gonadotropins like follicle stimulating hormone (Fsh) and luteinizing hormone (Lh) (7). The secretion of GnRH and gonadotropins are all controlled by the testicular steroids, including testosterone, estrogen inhibin and, activin all of which experience negative feedback inhibition during stress (8). Fsh directly stimulates the Sertoli cells supporting spermatogenesis, while Lh is required to stimulate the Leydig cells in the testes to secrete testosterone which acts on the Sertoli cells to aid in sperm production (9).

In our previous study we showed that probiotic strain *Lactobacillus gasseri* 505 (505), isolated from infant feces, and its synbiotic combination with *Cudrania tricuspidata* leaf extract (CT), one of the newly described plant-based prebiotics, improved antioxidative and anti-inflammatory activities and prevented the hepatotoxic effects associated with colorectal cancer in mice (10, 11). Moreover, our previous study determined that CT contained a variety of phenolic acids and flavonoids including predominant neo-chlorogenic acid, chlorogenic acid, caffeic acid, and quercetin-3-glucoside (12). In addition, enhancement of antioxidant activity of CT-supplemented fermented milk might be due to the generation of bioactive peptides during fermentation by 505 (12). However, the preventive effects of this synbiotic combination on male reproductive disorders resulting from chronic stress have not yet been evaluated.

Therefore, we aimed to investigate the protective effects of a synbiotic combination of 505 and CT on testicular tissues during chronic stress. This study used male mice as the model organism and in order to elucidate the mechanisms of this protection we performed histopathological examinations and determined the relative transcriptional profiles of several important testicular development markers. Since there are several side effects such as sexual dysfunction as well as headache, nausea, dizziness, and constipation after the clinical use of antidepressants (13, 14), it is necessary to develop the plant-based new therapeutics for the control of reproductive disorders associated with chronic stress.

## Materials And Methods

### Animals

**Figure 1:**
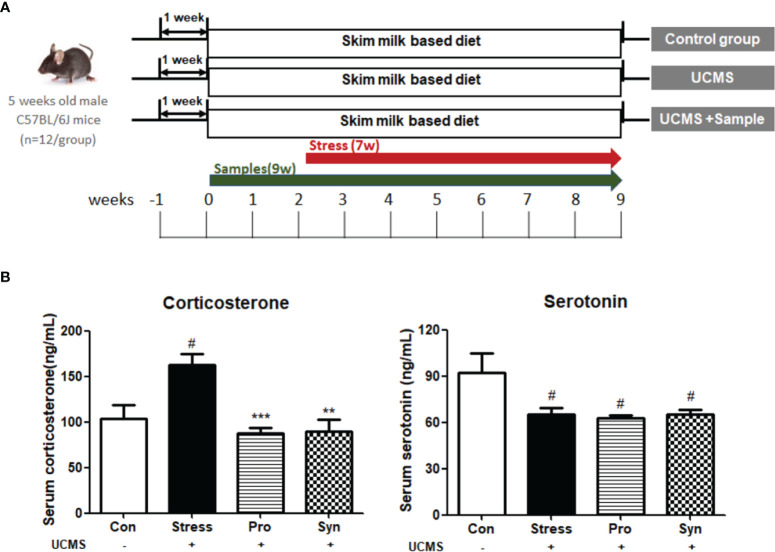
**(A)** Schematic overview of the animal experiments. **(B)** Effects of probiotics and synbiotics treatment on the serum concentrations of corticosterone and serotonin in mice during UCMS. Data are expressed as the mean ± SEM (n=12) from three independent experiments. ^#^Significant difference when compared to the Con group (^#^ *p* < 0.05, ^##^ *p* < 0.005, ^###^ *p* < 0.001). *Significant difference when compared to the Stress group (*p < 0.05, **p < 0.005, ***p < 0.001).

A total of 48 male C57BL/6J mice (8 weeks old) were purchased from Samtaco Bio Korea (Osan, Korea). The animals were maintained under a 12-hour light/dark cycle at 22 ± 2°C and RH 55% ± 5% for 7 days. Mice were given access to feed (AIN-76; DooYeol Biotech, Seoul, South Korea) and tap water *ad libitum* during the adaptation period. The mice were randomly assigned into one of the following four treatment groups (n = 12): (1) control group (Con); (2) unpredictable chronic mild stress (UCMS) group (Stress); (3) probiotics-treated group, treated with *Lactobacillus gasseri* 505 (505, 10^9^ CFU/kg/day) + UCMS (Pro); and (4) synbiotic-treated group, treated with a synbiotic combination of 505 and CT (1,500 mg/kg/day) + UCMS (Syn). After a 7-day adaptation period, each group was fed a skim milk-based diet supplemented with each treatment for 9 weeks. UCMS was applied for 7 weeks as previously described with minor modifications (15). The UCMS groups were exposed to repeated mild physical and psychological stressors each day including sleep cycle changes, wet bedding, tilted cages, changes in illumination, water deprivation, restraint, and cold-water baths in randomized orders. Control groups remained undisturbed during this period except for housekeeping procedures. The mice were euthanized at week 9 using CO_2_ inhalation and blood was collected immediately by cardiac puncture. The brain and testes tissues were dissected and stored at −20°C until further physiological analysis. The overall experimental design is described in **Figure 1A**. All the animal experiments of this study were approved by the Institutional Animal Care and Use Committee (IACUC) of Korea University (Seoul, South Korea, approval number KUIACUC-2016-182), and performed in accordance with the relevant guidelines and regulations.

### Preparation of the Probiotics and a Synbiotic Combination

The probiotic strain 505, originally isolated from human infant feces, was obtained from the Korean Culture Center of Microorganisms (Seoul, South Korea, KCCM 11766P). In our previous study, this strain was determined to possess various probiotic and functional properties including acid and bile tolerance, bacterial adhesion capacity, and anti-bacterial and cholesterol reducing abilities (11). The genetic information for this strain can be accessed with accession number KU517710 from NCBI GenBank (https://www.ncbi.nlm.nih.gov/genbank/). This strain was sub-cultured three times in de Man, Rogosa, and Sharpe (MRS) broth (Difco, MI, USA) at 37°C for 18 h, prior to use. Powdered prebiotic, CT, which selectively stimulates the growth and activity of 505 and releases a number of bioactive metabolites (11), was added to pre-warmed milk at a final concentration of 0.2% (g/g). This was then pasteurized at 85°C for 15 min and cooled to 41°C. This CT enriched pasteurized milk was inoculated into a 3% (v/v) suspension of 505 (approximately 10^7^ CFU/mL). This product was then incubated at 41°C for 40 h and all samples were stored at -20°C before use as a freeze-dried powder.

### Serum Analysis

Blood samples were immediately collected in BD Vacutainer^®^ SST™ II Advance (Becton Dickinson, NJ, USA) by cardiac puncture, and serum corticosterone and serotonin levels were measured as previously described (15). All measurements were made in triplicate.

### Histopathological Analysis

For histological analysis, mice testes were fixed in 4% paraformaldehyde in phosphate-buffered saline (PBS) (pH 7.4). After 24 h these fixed tissues were moved to 70% ethanol for 24 h and then dehydrated and embedded in Paraplast-Plus (Leica Microsystems, Wetzlar, Germany). Paraffin-embedded tissues were sectioned at 5 μm and stained with hematoxylin (Sigma-Aldrich, MO, USA) and eosin (Thermo Fisher, CA, USA). All images were captured using a Leica DM3000 microscope (Leica Microsystem Corp, Wetzlar, Germany).

### Quantitative Reverse-Transcription PCR

The expression of mRNA was evaluated with quantitative reverse transcription PCR (qRT-PCR). Total RNA was extracted from frozen tissues as previously described (15). Relative gene expression was then calculated using the comparative threshold cycle method, and values were normalized against those for glyceraldehyde-3-phosphate dehydrogenase (*GAPDH*), which acted as the internal control. The primer sequences used for qRT-PCR are listed in **Table 1**.

**Table 1:** Details of the primers used in this study.

Gene Symbol	Forward primer (5’→3’)	Reverse primer (5’→3’)
GnRH	AGCACTGGTCCTATGGGTTG	GGGGTTCTGCCATTTGATCCA
GnRHr	TGCTCGGCCATCAACAACA	GGCAGTAGAGAGTAGGAAAAGGA
Fshb	GCCATAGCTGTGAATTGACCA	AGATCCCTAGTGTAGCAGTAGC
Lhb	CTGAGCCCAAGTGTGGTGTG	GACCATGCTAGGACAGTAGCC
Adam5	AGGAGAATCTGTGGCAATGG	CCGTGCAATCTTGACTACAGC
Adam29	CCATGAATGTCCAGATGATGC	TTGCCTACAGTGCTCATTGC
Spam1	GAATGGAGGCCTACCTGGTT	CTTCCTTCCTGCCTCTTCAA
Tcfap2c	AGAGGAGGTGCAGAATGTGG	CAGGGACTGAGCAGAAGACC
Lhr	CGCCCGACTATCTCTCACCTA	GACAGATTGAGGAGGTTGTCAAA
Egfr	GGGGATGTGATCATTTCTGG	GCCTTGCAGTCTTTCTCAGC
Inha	GTCTCTGCTGCTCCTTTTGC	GGAATAGAGCCTTCACCTTGG
StAR	TCTGCTTGGTTCTCAACTGG	TTCTGCATAGCCACCTCTCC
Srd5a3	CCGCCCATCAGTATAAATGC	CTCGAACCAGTCTCCAAAGG
Cyp19a1	TGTTGTGGACTTGGTCATGC	TGGGCTTAGGGAAGTACTCG
Gapdh	GACGGCCGCATCTTCTTGT	CAGTGCCAGCCTCGTCCCGTACAA

### *In Situ* Hybridization Analysis

*In situ* hybridization analysis was performed as the methods of previous studies (16, 17) to localize *Adam5*, *Adam29*, *Spam1*, *Lhr*, *Egfr*, and *StAR* in section of the testes.

### Statistical Analysis

SPSS software version 22.0 (IBM, Chicago, IL, USA) was used for statistical analyses. All data are shown as the mean ± standard error standard error of the mean (SEM). Statistical significance for between-group differences was assessed using an independent-sample t-test and a two-way ANOVA. Significant difference between Con and Stress groups as well as significant difference of sample treated groups compared to the Stress group were statistically analysed by an independent-sample t-test (at a significance level of *p* < 0.05). A two-way ANOVA (*p* < 0.05) was carried for the statistical analysis of data on changes by stress and treatment of samples. Moreover, Tukey’s *post hoc* test was used after ANOVA.

## Results

### Effects of Probiotics and Synbiotics Treatment on Serum Stress Markers and Testicular Histopathology

**Figure 2:**
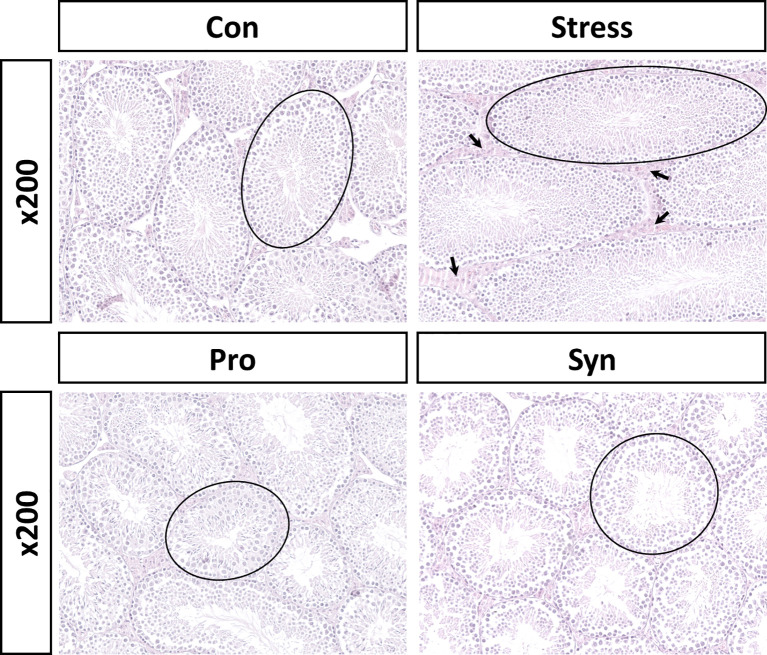
Representative histological sections from mice testes stained with hematoxylin and eosin (calibration bar = 200 μm). Area of seminiferous tubules (black circle), Leydig cell area (black arrow).

To determine the physiological response to chronic stress and the effect of probiotics and synbiotics treatment, we evaluated the expression of corticosterone and serotonin and performed completed testicular histopathological analyses (**Figures 1B**, **2**). Exposure to UCMS significantly increased the serum corticosterone level compared to the Con group. However, the serum levels of corticosterone were significantly lower in the Pro and Syn groups compared to the Stress group. The serum serotonin levels dropped significantly when exposed to UCMS, but there were no significant changes in this biomarker following Pro or Syn treatment. A two-way ANOVA resulted that the exposure to stress had a significant effect on serum corticosterone and serotonin levels, however treatment of samples showed a significant effect on serum corticosterone level not serotonin level. The histopathological changes indicated that the tubular area of the seminiferous tubule sections and the Leydig cell area were extensively increased in the Stress group compared to the Con group. However, these parameters recovered back to normal in the Pro and Syn groups.

### Effects of Probiotics and Synbiotics Treatment on the HPG Axis During UCMS

**Figure 3:**
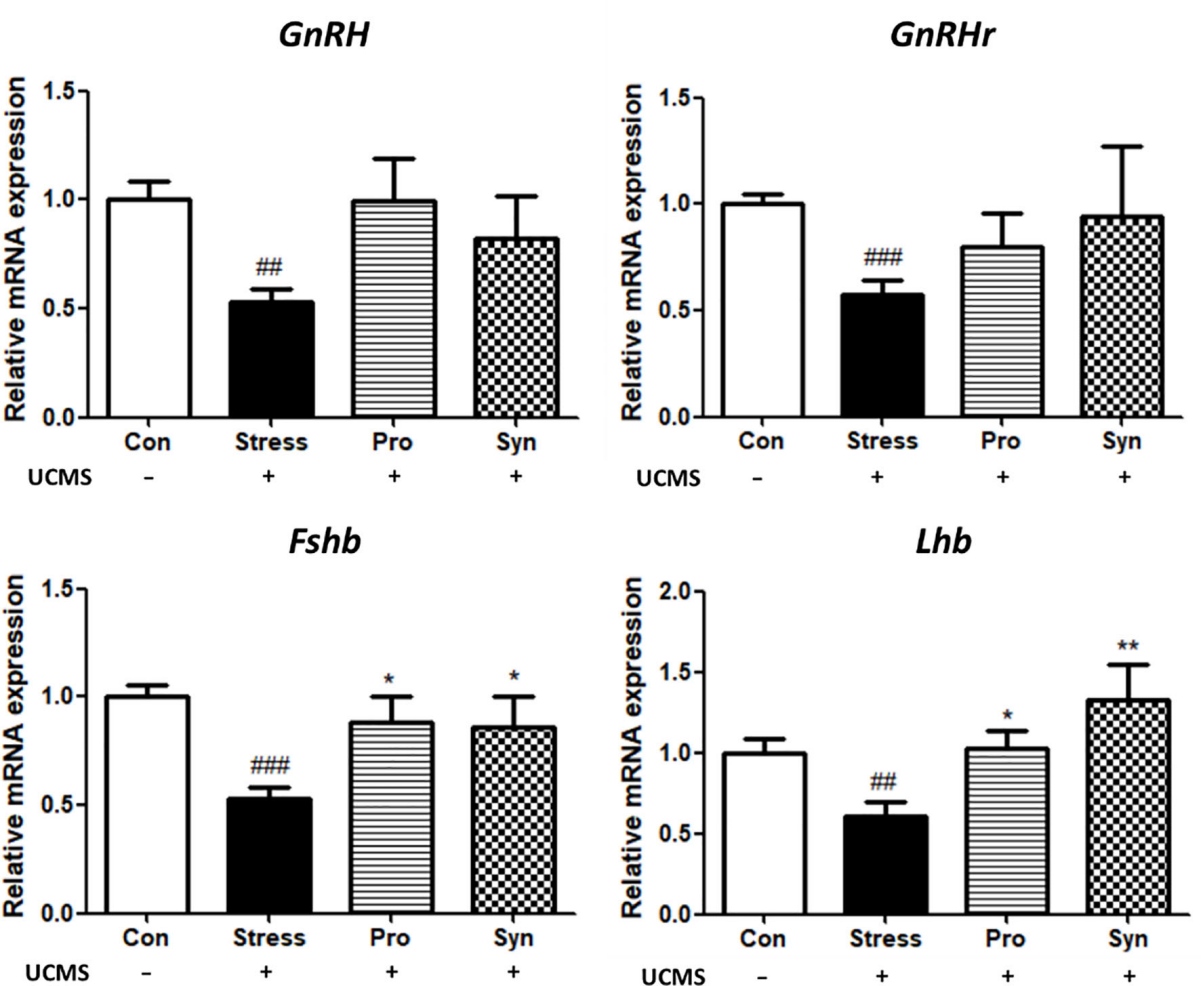
Effects of probiotics and synbiotics treatments on the transcription of genes related to the HPG axis in mouse brains during UCMS. Data represents the relative expression of *GnRH*, *GnRHr*, *Fshb*, and *Lhb* compared to *Gapdh*. Data are expressed as the mean ± SEM (n=12) from three independent experiments. ^#^Significant difference when compared to the Con group (^#^ *p* < 0.05, ^##^ *p* < 0.005, ^###^ *p* < 0.001). *Significant difference when compared to the Stress group (*p < 0.05, **p < 0.005, ***p < 0.001).

qRT-PCR was used to determine the effect of probiotics and synbiotics treatment on the transcription of those genes associated with the HPG axis (**Figure 3**). Exposure to UCMS significantly down-regulated the expression of gonadotropin-releasing hormone (*GnRH*) and its receptor (*GnRHr*) at the transcript level, while treatment with the probiotics or and synbiotic compounds inhibited the UCMS-induced repression of these genes. The transcription of gonadotropins such as follicle stimulating hormone beta (Fshβ) and luteinizing hormone beta (Lhβ) were also significantly decreased during chronic stress. With the transcription of these genes significantly upregulated in the Pro and Syn groups compared to the Stress group, returning their expression to normal ranges comparable to those described for the Con group. Similarly, the result of ANOVA showed a significant effect of stress exposure (*p* < 0.05) to the expression of those genes, however treatment of samples had a significant effect (*p* < 0.05) on the expression of *Fshβ* and *Lhβ*.

### Effects of Probiotics and Synbiotics Treatment on the Expression of Testicular Development-Related Genes During UCMS

**Figure 4:**
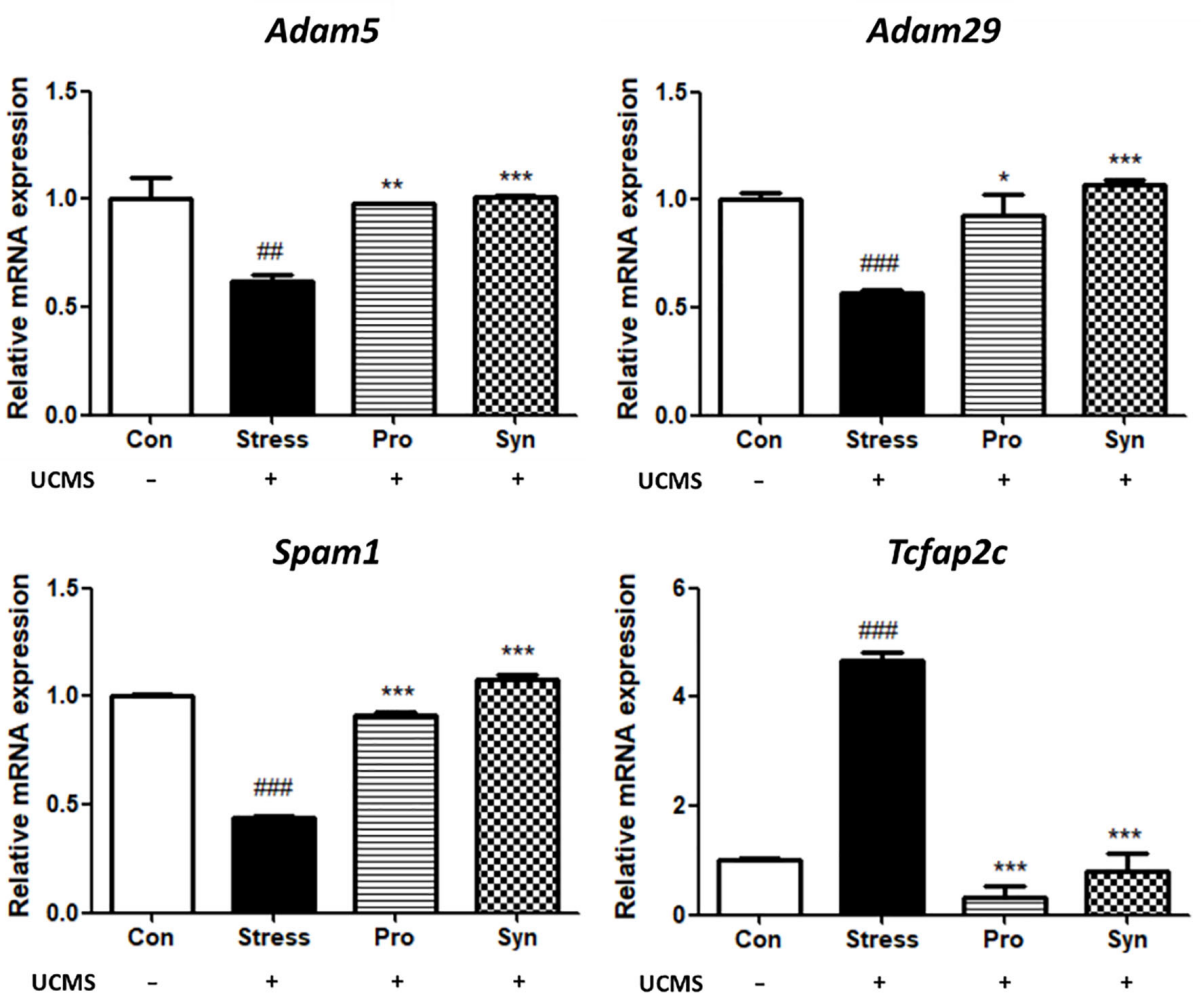
Effects of probiotics and synbiotics treatments on the transcription of testicular development genes in mice testes under UCMS. Data represents the relative expression of *Adam5*, *Adam29*, *Spam1*, and *Tcfap2c* compared to *Gapdh*. Data are expressed as the mean ± SEM (n=12) from three independent experiments. ^#^Significant difference compared to the Con group (^#^ *p* < 0.05, ^##^ *p* < 0.005, ^###^ *p* < 0.001). *Significant difference compared to the Stress group (*p < 0.05, **p < 0.005, ***p < 0.001).

The changes in the transcription of the testes development-related genes in mice with UCMS are shown in **Figure 4**. The Stress treated group demonstrated significant differences in the expression of a disintegrin and metalloproteinase 5 (*Adam5*), a disintegrin and metalloproteinase 29 (*Adam29*), Sperm adhesion molecule 1 (*Spam1*), transcription factor AP-2gamma (*Tcfap2c*) when compared to the Con group. However, treatment with probiotics or synbiotics inhibited these UCMS-induced changes. The Syn group in particular showed significant recovery compared with the Stress and Pro groups. Moreover, a two-way ANOVA reveled that stress exposure (*p* < 0.05) and sample treatment (*p* < 0.05) both a statistically significant effect on the expression of testicular development-related genes.

### Effects of Probiotics and Synbiotics Treatment on the mRNA Expression of Steroidogenesis Genes and Growth Factors During UCMS

**Figure 5:**
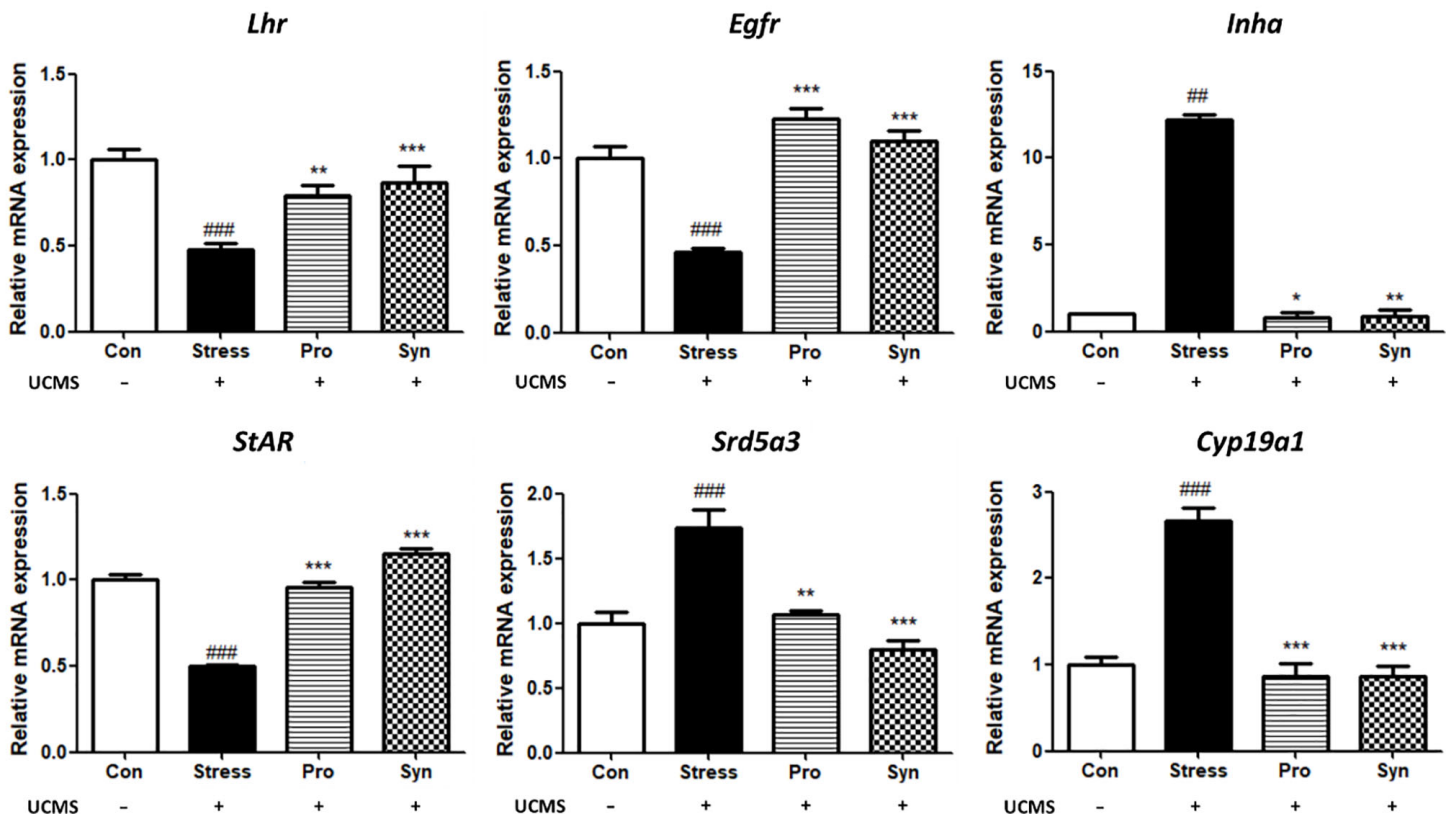
Effects of probiotics and synbiotics treatments on the transcription of spermatogenesis and growth factor related genes in mice testes under UCMS. Data describes the relative expressions of *Lhr*, *Egfr*, *Inha*, *StAR*, *Srd5a3*, and *Cyp19a1* compared to *Gapdh*. Data represent the mean ± SEM (n=12) from three independent experiments. ^#^Significant difference compared to the Con group (^#^ *p* < 0.05, ^##^ *p* < 0.005, ^###^ *p* < 0.001). *Significant difference compared to the Stress group (*p < 0.05, **p < 0.005, ***p < 0.001).

The effects of probiotics and synbiotics treatment on the expressions of steroidogenesis- and growth factor-related genes during UCMS are shown in **Figure 5**. In the Stress group, the Lh receptor (*Lhr*), epidermal growth factor receptor (*Egfr*), and steroidogenic acute regulatory protein (*StAR*) were all significantly downregulated. However, inhibin alpha (*Inha*), steroid 5 alpha-reductase 3 (*Srd5a3*), and cytochrome P450 family 19 subfamily A member 1 (*Cyp19a1*) were all significantly upregulated in stressed mice when compared to non-stressed mice. In the sample treatment groups, Pro and Syn, the abnormal expression of these genes was inhibited and their expression profiles returned to normal levels, similar to those observed in the control group. This was especially evident in *Lhr*, *Inha*, and *Srd5a3* expression and these changes were more significant in the Syn group. In addition, statistical analysis with a two-way ANOVA indicated significant effects of stress exposure (*p* < 0.05) and sample treatment (*p* < 0.05) on the expression of steroidogenesis genes and growth factors.

### Localization of Male Reproductive Transcripts in Testicular Tissues

**Figure 6:**
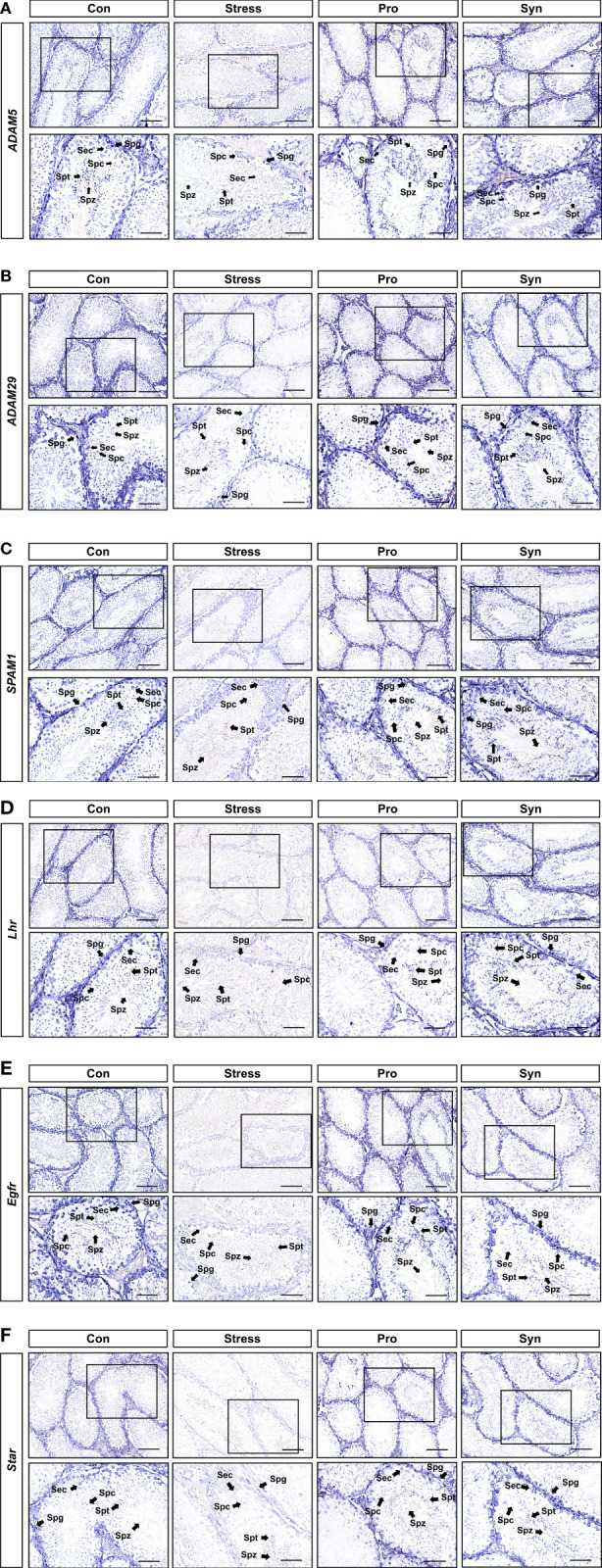
*In situ* hybridization of *Adam5*, *Adam29*, *Spam1*, *Lhr*, *Egfr*, and *StAR* **(A–F)** in mice testes. mRNA expression was analyzed using cross-sections from mice testes and antisense or sense target gene cRNA probes. L, Leydig cells; Spc, spermatocytes; Spg, spermatogonia; Spz, spermatozoa. Scale bar represents 10 μm.

A detailed analysis of the male reproductive system-related genes (i.e., *Adam5*, *Adam29*, *Spam1*, *StAR*, *Egfr*, and *Lhr*) in the testes following UCMS treatment was performed using *in situ* hybridization (**Figure 6**). The expression levels of these genes were reduced in the spermatocytes (Spc), spermatogonia (Spg), spermatids (Spt), and spermatozoa (Spz) of mice treated with UCMS when compared to the control group. Conversely, higher expression levels for each of these genes were observed following probiotics or synbiotics treatment. The expression of *Adam5*, *Spam1*, and *Lhr* showed the greatest improvement in the Syn treated group. As with the results of the qRT-PCR, *in situ* hybridization revealed that chronic stress had a negative effect on the expression of the testicular reproductive genes, including testicular development markers, steroidogenesis markers, and hormone/growth markers. Furthermore, these results indicate that the administration of probiotic strain 505 and its synbiotics containing CT could prevent the male reproductive dysfunction associated with chronic stress.

## Discussion

In this study, a synbiotic combination made up of *Lactobacillus gasseri* 505 and *Cudrania tricuspidata* leaf extracts could provide a protective effect for testicular development and function during chronic stress. UCMS exerts a negative effect on the expression of reproductive-related genes in mice brains and testes, leading to deteriorations in the male reproductive system, including abnormal testicular development, steroidogenesis, transcription, and hormone/growth factor expression. In contrast, UCMS-induced reproductive dysfunctions were improved *via* the HPG axis following treatment with probiotic strain 505 and its synbiotics containing CT. These preventive effects may be the result of the probiotic strain 505 or the phenolic compounds and peptides derived from the fermentation of CT-supplemented milk by 505 (12). In our previous study, 505 was shown to exhibit acid and bile tolerance, adhesion capacity, antibacterial activity, and cholesterol-reducing ability (11). Moreover, CT contains appreciable levels of several phenolic compounds, including chlorogenic acid, caffeic acid, quercetin-3-glucoside, and 3,4-dihydroxy-hydrocinnamic acid, which have all been described as exerting various antioxidant properties (12). In addition, various bioactive peptides (i.e., antimicrobial, antihypertensive, and antioxidative activities and ACE inhibitory activity) have been identified following the fermentation of CT-supplemented milk with 505 (12). These bioactive compounds and probiotic strain impacted the preventive effects for chronic stress-induced testicular dysfunctions in their synbiotic combination.

The histological evaluation of the testes revealed that UCMS induced the disruption of the multilayered epithelial skeleton and diminished the number of spermatozoa in the seminiferous tubules. A significant increase in the extent of cellular damage, and thus testicular damage, was observed in the testicular sections of mice with UCMS when compared to the Con group. Conversely, treatment with probiotics or synbiotics showed recovery in the seminiferous tubules structure with these structures almost returning to their pre-stressed condition. These results indicate that the administration of 505 and/or its synbiotics improved stress response and reduced cellular damage in the testes. Several researchers have also determined that the male reproductive capacity was regularly decreased due to stress, which supports our study (18, 19). Moreover, treatment of 505 and synbiotics prevent UCMS-induced downregulation of *GnRH* and *GnRHr* and their downstream gonadotropins, which may help to retain reproductive function during chronic stress. Repetative exposure to stress is biomedically critical in male reproductive disorder which is supported by previous studies that chronic stress increased the activity of the hypothalamus-pituitary-adrenal (HPA) axis and suppressed sperm motility by disturbing HPG axis activity (20, 21). The secretion of GnRH and gonadotropins are all controlled by the testicular steroids, including testosterone, estrogen inhibin and, activin all of which experience negative feedback inhibition during stress (8). GnRH is a tropic peptide hormone synthesized and released from GnRH neurons within the hypothalamus, and constitutes the initial step in the HPG axis leading to the release of Fsh and Lh from the anterior pituitary (22). Fsh, a glycoprotein polypeptide hormone, enhances the production of androgen binding protein in the Sertoli cells of the testes by binding to the Fsh receptors on their membranes, and is critical for the initiation of spermatogenesis (23). Lh stimulates Leydig cells to produce testosterone (24), since Lh regulates the expression of 17β-hydroxysteroid dehydrogenase which converts androstenedione to testosterone, an androgen that exerts both endocrine and intra-testicular effects on spermatogenesis (25). Besides, adam family proteins expressed in male reproductive tissues undergo unique processing during sperm maturation and are located on the surface of the sperm heads (26). *Adam5* is known to play a particularly important role in fertilization, while *Adam29* is involved in transducing cellular signals related to maturation of testicular cells (27). *Spam1* encodes an enzyme located on the sperm surface and inner acrosomal membrane. This multifunctional protein is a hyaluronidase that enables sperm to penetrate the hyaluronic acid-rich cumulus cell layer surrounding the oocyte, a receptor that plays a role in hyaluronic acid induced cell signaling, and a receptor that is involved in sperm-zona pellucida adhesion (28). Moreover, Hoei-Hansen *et al.* (29) reported that *Tcfap2c* is involved in the undifferentiated phenotype in germ cells and could be a marker of testicular tumors.

In our study, exposure to UCMS downregulated a number of genes related to testicular development which could result in testicular dysfunction. However, treatment with either the probiotic strain 505 or its synbiotics with CT, especially the synbiotics, prevent the UCMS-induced changes in the expressions of these genes which may lead to protection of testicular involution. In addition, the results for the preventive effects of 505 and synbiotics on steroidogenesis were in agreement with a study conducted by Kushwaha and Gupta (30) which showed that chronic stress had a negative effect on the male reproductive system *via* the HPG axis, effected by endocrine activity and Lh secretion. Lh interacts with its receptor Lhr and this activation is critical for spermatogenesis while Egfr is the prototypical member of its family of membrane associated intrinsic tyrosine kinase receptors, which are required for optimal embryonic testes growth (31). Aberrant Egfr activation is a significant factor in the development and progression of multiple cancers (32). Inhibin encoded by *Inha* has been shown to be a negative regulator of gonadal stromal cell proliferation and suppresses Fsh secretion by acting directly at the pituitary (33). The protein encoded by *StAR* plays a crucial role in the acute regulation of steroid hormone synthesis by enhancing the conversion of cholesterol into pregnenolone. Mutations in the *StAR* locus result in congenital lipoid adrenal hyperplasia, in which steroid hormone biosynthesis is severely compromised (34). The testes-derived hormone, testosterone must be converted into dihydrotestosterone (DHT), and this conversion is catalyzed by the microsomal enzyme encoded by *Srd5a3*. *Srd5a3* activity is a late stage event in male sexual development, that requires the correct developmental interpretation of both genetic and hormonal signals (35). The overexpression of *Srd5a3* increases the levels of DHT, which causes hair loss and prostate diseases including benign prostatic hyperplasia and prostate cancer (36). Testosterone is also converted to estradiol by *Cyp19a1* activating the nuclear estrogen receptor (37). *Cyp19a1* is highly expressed in the undifferentiated gonads and is upregulated during initial male sexual differentiation events (38). These results indicate that treatment with 505 or its synbiotics could prevent the abnormal hormonal biosynthesis associated with UCMS-induced testicular dysfunctions *via* transcriptional regulation of key developmental factors. Taken together, current study demonstrated that administration of a synbiotic combination of 505 and CT attenuated the UCMS-induced alteration in testicular development- and steroidogenesis-related genes, which was also confirmed with *in situ* hybridization analysis. Consequently, this study suggested that this novel synbiotics could act as a natural protective agent for male fertility during chronic stress.

## Data Availability Statement

The original contributions presented in the study are included in the article/supplementary material. Further inquiries can be directed to the corresponding authors.

## Ethics Statement

The animal study was reviewed and approved by the Institutional Animal Care and Use Committee (IACUC) of Korea University (Seoul, South Korea, approval number KUIACUC-2016-182).

## Author Contributions

NSO and SHK conceived and designed the research, and revised the manuscript. JYJ and WL conducted the experiments, analyzed data, and wrote the manuscript. YJS and JH conducted the experiments. All authors read and approved the final manuscript.

## Funding

This work was supported by Korea Institute of Planning and Evaluation for Technology in Food, Agriculture and Forestry (IPET) through High Value-added Food Technology Development Program, funded by Ministry of Agriculture, Food and Rural Affairs (MAFRA) (321036-5) and Basic Science Research Program through the National Research Foundation of Korea (NRF) funded by the Ministry of Education (NRF-2020R1F1A1055542).

## Conflict of Interest

The authors declare that the research was conducted in the absence of any commercial or financial relationships that could be construed as a potential conflict of interest.

## Publisher’s Note

All claims expressed in this article are solely those of the authors and do not necessarily represent those of their affiliated organizations, or those of the publisher, the editors and the reviewers. Any product that may be evaluated in this article, or claim that may be made by its manufacturer, is not guaranteed or endorsed by the publisher.
